# Photon-Counting Detector CT Virtual Monoengergetic Images for Cochlear Implant Visualization—A Head to Head Comparison to Energy-Integrating Detector CT

**DOI:** 10.3390/tomography8040136

**Published:** 2022-06-21

**Authors:** Stephan Waldeck, Daniel Overhoff, Leona Alizadeh, Benjamin V. Becker, Matthias Port, Matthias F. Froelich, Marc A. Brockmann, Sven Schumann, Thomas J. Vogl, Stefan O. Schoenberg, Sandra Schmidt

**Affiliations:** 1 Department of Diagnostic and Interventional Radiology and Neuroradiology, Bundeswehr Central Hospital Koblenz, Rübenacher Straße 170, 56072 Koblenz, Germany; daniel.overhoff@umm.de (D.O.); leona.alizadeh@outlook.de (L.A.); benjamin3becker@bundeswehr.org (B.V.B.); 2 Institute of Neuroradiology, University Medical Centre, Johannes Gutenberg University Mainz, Langenbeckstraße 1, 55131 Mainz, Germany; mbrockma@uni-mainz.de; 3 Department of Radiology and Nuclear Medicine, University Medical Centre Mannheim, Medical Faculty Mannheim, Heidelberg University, Theodor-Kutzer-Ufer 1–3, 68167 Mannheim, Germany; matthias.froelich@medma.uni-heidelberg.de (M.F.F.); stefan.schoenberg@umm.de (S.O.S.); 4 Bundeswehr Institute of Radiobiology, Neuherbergstraße 11, 80937 München, Germany; matthiasport@bundeswehr.org; 5 Institute of Anatomy, University Medical Center, Johannes Gutenberg-University Mainz, 55131 Mainz, Germany; sven.schumann@uni-mainz.de; 6 Department of Diagnostic and Interventional Radiology, University Hospital Frankfurt, 60590 Frankfurt am Main, Germany; t.vogl@em.uni-frankfurt.de; 7 Department of ENT Surgery, Bundeswehr Central Hospital Koblenz, Rübenacher Straße 170, 56072 Koblenz, Germany; drsandraschmidt@googlemail.com

**Keywords:** cochlear implant, spatial resolution, high-resolution computed tomography, metal artifacts, virtual monoenergetic imaging, photon-counting detector CT, energy-integrating detector CT, cadaver study

## Abstract

Cochlear implants (CIs) are the primary treatment method in patients with profound sensorineural hearing loss. Interpretation of postoperative imaging with conventional energy-integrating detector computed tomography (EID-CT) following CI surgery remains challenging due to metal artifacts. Still, the photon-counting detector (PCD-CT) is a new emerging technology with the potential to eliminate these problems. This study evaluated the performance of virtual monoenergetic (VME) EID-CT images versus PCD-CT in CI imaging. In this cadaveric study, two temporal bone specimens with implanted CIs were scanned with EID-CT and PCD-CT. The images were assessed according to the visibility of interelectrode wire, size of electrode contact, and diameter of halo artifacts. The visibility of interelectrode wire sections was significantly higher when reviewing PCD-CT images. The difference in diameter measurements for electrode contacts between the two CT scanner modalities showed that the PCD-CT technology generally led to significantly larger diameter readings. The larger measurements were closer to the manufacturer’s specifications for the CI electrode. The size of halo artifacts surrounding the electrode contacts did not differ significantly between the two imaging modalities. PCT-CT imaging is a promising technology for CI imaging with improved spatial resolution and better visibility of small structures than conventional EID-CT.

## 1. Introduction

Cochlear implants (CIs) are the treatment method of choice in patients with profound sensorineural hearing loss [1]. Operative protocols require preoperative imaging to screen for any cochlear anomalies such as cochlear malformation or obliteration, which may impede CI placement. Optimal CI function is dependent on a variety of factors including, but not limited to cause, duration, and extent of hearing loss and correct electrode placement [2].

Assurance of the latter is primarily based on postoperative imaging following CI placement. Currently, no clinical guidelines exist for postoperative imaging of the temporal bone, so surgeons’ choices are generally conventional Stenver’s projection radiography, cone-beam computed tomography (CBCT), and computed tomography (CT) [3,4,5]. Conventional X-ray remains a quick, practical, and low-cost evaluation method but does not allow assessment of the structural relationship between the CI electrode and the cochlea [6,7]. CBCT allows for low-dose postoperative 3D evaluation of electrode insertion with low metal artifacts and the possibility of automated cochlea segmentation [3,8]. High-resolution computed tomography (HR-CT) is a commonly used and well-established imaging alternative for postoperative CI evaluation that provides good spatial resolution and improved diagnostic accuracy [9]. However, due to metal artifacts, interpretation of HR-CT postoperative imaging following CI placement remains challenging despite the implementation of technological advances [10,11].

Current approaches to reducing metal artifacts include the application of virtual monoenergetic (VME) imaging for dual-energy CT. Unfortunately, even though the ultra-high-resolution (UHR) mode of newer generation energy-integrating detector (EID) CT scanners has improved spatial resolution of fine structures like the temporal bones, further refinement is limited by the detector’s characteristics [12]. The detector pixel size of 0.5 to 0.625 mm at the isocenter of the latest generation CT scanners remains the main limitation of further spatial resolution improvements [13].

In contrast, photon-counting detector (PCD) CT is a new emerging technology with the potential to eliminate these problems. PCD-CT converts every X-ray photon into a charge cloud which can be measured. As a result, every photon and the energy of every photon can be counted, and thus PCD-CT provides spectral CT information. This reduction in detector pixel size at the isocenter leads to multiple clinical benefits such as higher spatial resolution and reduced signal-to-noise and contrast-to-noise ratios [14,15].

The PCD-CT system also includes a UHR acquisition mode, especially for high-contrast and high-resolution structures such as the temporal bone. Several animal, retrospective, and cadaveric studies have proven the utility of PCT-CT technology in temporal bone imaging [16,17,18]. However, the potential role of PCD-CT in postoperative CI imaging has not yet been investigated.

The study aim was, therefore, to evaluate the performance of UHR PCD-CT images of CI electrodes and compare the results with those of standard CT (UHR EID-CT) acquisitions.

## 2. Materials and Methods

The human bodies in this study were donated to science at the Institute of Anatomy, University Medical Center of the Johannes Gutenberg-University Mainz, Mainz, Germany, according to the Department’s Donation Program. Donations were provided exclusively by the person’s own last will and written consent. Since this is the highest ethical donation, no further vote of an Ethics Committee was necessary. The present study was conducted according to the guidelines of the Declaration of Helsinki and within the parameters of the written permission that the Institute of Anatomy received from the body donors during their lifetime.

**Figure 1:**
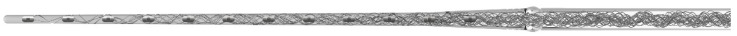
Close-up view of a MED-EL standard electrode array with electrode contacts and connecting wires.

An experienced neurotologist performed cochlear implant placement surgery in two cadaveric temporal bone specimens using one SYNCHRONY Standard (MED-EL, Innsbruck, Austria) CI and one SYNCHRONY Medium (MED-EL, Innsbruck, Austria) CI. The CI electrode array (Figure 1) is the part of the CI that is inserted into the cochlea during cochlear implantation surgery. The electrode array consists of a silicone carrier with twelve integrated electrode contacts. The electrode contacts are made of a platinum–iridium alloy and are connected either by straight or wave-shaped wires. The highest number of wires is located in the initial part of the electrode array and decreases with each subsequent electrode contact en route to the tip of the electrode array.

To ensure the correct electrode placement in the specimens we used EID and PCD-CT systems and compared the results. The detailed setup description of CT scanners has been described in previous articles [14,19].

The EID-CT utilized a standard latest generation dual source CT scanner (3rd Generation Dualsource CT; Somatom Force, Siemens Healthineers, Erlangen, Germany). The PCD-CT was a newly developed dual source CT scanner (Naeotom alpha, Siemens Healthineers, Erlangen, Germany).

The following scan protocol was used for the dual source CT scanner: dual energy (DE) 80 kV/150 kV tin filtration; 29 mA, 4.16 CTDI, 192 × 0.6 mm collimation, 0.6 mm reconstruction thickness.

The following scan protocol was used for the PCD-CT: 120 kV, 32 mA, 5.44 CTDI, 144 × 0.4 mm collimation, and 0.4 reconstruction thickness.

The images were reconstructed using VME technology and analyzed at the following kiloelectron-volt (keV) settings: 80 keV, 100 keV, 120 keV, 140 keV, 160 keV, 180 keV, and 190 keV.

Two experienced neuroradiologists then assessed the CI electrodes’ reconstructed VME images at each keV level for both imaging modalities by examining each contact separately. The neuroradiologists first measured the diameter of each contact and the diameter of the surrounding halo for all twelve electrode contacts. Secondly, they rated the visibility of interelectrode wire on a 0–1 Likert scale (0 = interelectrode wire not visible; 1 = visible interelectrode wire).

### Statistical Analyses

All statistical analyses were performed with SPSS (IBM SPSS Statistics, version 20.0 for Macintosh; SPSS, Inc., Chicago, IL, USA). The McNemar test was used to compare the visibility of the interelectrode wire. Data on the diameter of contacts and halo artifacts were compared with a paired samples t-test. A *p*-value < 0.05 was considered significant.

## 3. Results

### 3.1. Visibility of Wire between Individual Electrode Contacts

**Figure 2:**
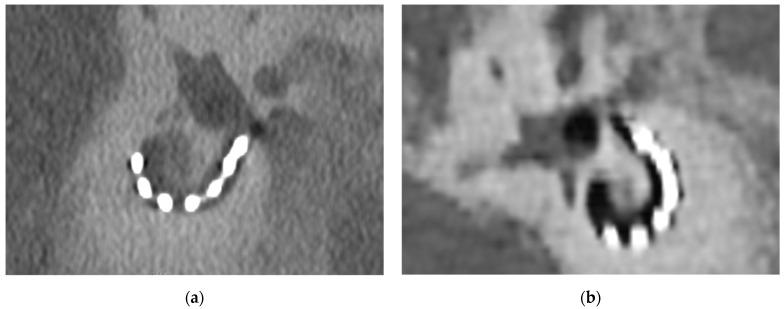
Comparison of the visibility of an implanted CI and the surrounding temporal bone. (**a**) PCD-CT image of CI following implantation surgery at 160 kV; (**b**) EID-CT image of CI following implantation surgery at 160 kV. The metal-to-bone contrast is better for the PCD-CT images.

The visibility of the connecting wire between individual electrode contacts was assessed at *n* = 168 total sites. With EID-CT, the interelectrode wire was visible in 114 (67.9%) cases and not visible at 54 (32.1%) sites. The PCD-CT afforded visibility in 137 (81.5%) sites whereas the interelectrode wire was not visible in 31 (18.5%) cases. The McNemar test showed a statistically significant difference (*p* < 0.001) between the two imaging modalities. The visibility of interelectrode wire sections was significantly higher when reviewing PCD-CT images. These results can be accounted for by the higher resolution of the PCD-CT technology (Figure 2).

### 3.2. Diameter Measurements for Electrode Contacts

**Figure 3:**
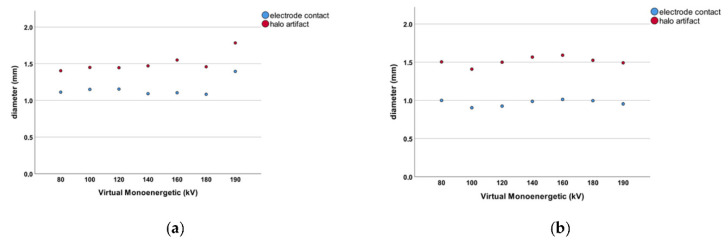
(**a**) Dot chart for the PCD-CT measurements. The blue dots represent the diameter of the individual electrode contacts for each VME setting. The red dots represent the diameter of the respective halo artifact for that VME increment. (**b**) Dot chart for the EID-CT measurements. The blue dots represent the diameter of the individual electrode contacts for each VME setting. The red dots represent the diameter of the respective halo artifact for that VME increment.

We measured the diameter of all contacts (*n* = 168) at the aforementioned VME settings. The electrode contacts measured 1.156 ± 0.193 mm in diameter when PCD-CT images were reviewed. For conventional EID-CT, the mean diameter of electrode contacts was 0.968 ± 0.111 mm (Figure 3). This difference was statistically significant (*p* < 0.001). The difference in diameter measurements for electrode contacts between the two CT scanner modalities shows that the PCD-CT technology leads to larger diameter readings in general. The results of the PCD-CT measurements were thus closer to the 1.3 × 1.3 mm original electrode contact diameter as specified by the manufacturer.

### 3.3. Halo Artifacts for Electrode Contacts

The size of halo artifacts surrounding all electrode contacts (*n* = 168) was measured. The halo artifact diameter was 1.510 ± 0.245 mm in PCD-CT scans compared to 1.513 ± 0.220 mm measured with conventional EID-CT technology (Figure 3). These measurements did not reach statistical significance (*p* = 0.931). There was no difference in halo artifact diameter between the EID- and PCD-CT imaging modalities.

## 4. Discussion

Metal artifacts continue to present a challenge when interpreting CT images despite technological advances such as VME reconstruction algorithms [10,11,20]. Postoperative imaging following CI implantation surgery is a crucial element in ensuring correct CI placement and proper CI function [2]. However, conventional EID-CT has limited spatial resolution for temporal bone even in UHR mode, and the metal artifacts that accompany CIs further reduce the image quality.

We aimed to evaluate the differences in spatial resolution and metal artifacts between PCD-CT and conventional EID-CT for VME images. The results of our study suggest that PCD-CT allows for greater visibility of CI electrodes without changes in metal artifact diameter.

To the best of our knowledge, this is the first study examining the use of a PCD-CT scanner for CI imaging. The innovative element in PCD-CT scanners is their unique ability to convert X-rays into electrical current directly, instead of first generating visible light. Specific CT detectors were designed to convert each photon’s energy into a measurable charge cloud. Direct conversion has many benefits such as improved image quality, better contrast, and increased image sharpness. Due to the novelty of the technology, with the first commercialized PCD-CT scanner gaining clearance from the United States Food and Drug Administration on September 30 of 2021, there is only a limited amount of literature available [21].

Among these studies, several have focused on the temporal bone, as it remains one of the smallest bony anatomical structures in the human body. In line with the findings of this study, Zhou et al., (2018) found that PCD-CT technology increased in-plane resolution and had significantly less image noise compared to conventional EID-CT-generated images in a cadaveric study of ten human temporal bones. The three blinded neuroradiologists preferred the PCD-CT images over the EID-CT scans and ranked overall image quality significantly higher for the novel technology [16]. Phantom and cadaveric studies showed that images generated by PCD-CT in UHR acquisition mode with an additional tin filter resulted in a dose reduction of 83% for temporal bone scans compared to conventional EID-CT. Even with the massive reduction in clinical radiation dose, both imaging modalities showed similar visualization capabilities regarding structures of the inner ear such as the incudomalleolar joint [17]. Leng et al., (2016) published the preliminary results of studies involving several phantom and cadaveric test specimens for PCD-CT imaging in UHR acquisition mode. A 29% noise reduction was observed when implementing the PCD-CT technology for temporal bone scanning compared to traditional EID-CT imaging [13]. The significantly improved visibility of interelectrode CI wire with PCD-CT found in this study can be attributed to both the higher spatial resolution and better metal-to-bone contrast. This has also been confirmed for studies outside of the temporal bone [22,23].

Hard device failures represent the most common indication for CI reimplantation surgery [24,25]. The better visibility of the interelectrode wire holds the potential for narrowing down the reasons for the diagnosis of device failures in a clinical workup can exclude the interelectrode wire as the reason for this failure. Better visibility of the parts of the electrode may also contribute to an optimized diagnosis of other factors for device failure such as insertion-related factors including tip fold over or late, electrode-related complications such as migration.

In this study, the increased metal-to-bone contrast led to more accurate measurements in terms of CI electrode contacts, when using the PCD-CT. However, there was no significant difference in halo artifact diameter between the two imaging modalities.

Although many articles on PCD-CT scanners quote reduced metal artifacts as one of the main benefits of the technology there is currently very limited evidence on the actual quantitative reduction of metal artifacts. Zhou et al., (2019) studied images of a phantom 3D-printed spine with pedicle screws generated by PCD-CT and EID-CT scanners, followed by clinical images of patients with prostheses of the spine, shoulder, wrist, elbow, and ankle. A highly significant reduction in metal artifact size was noted for PCD-CT images compared to conventional EID-CT images [22]. The results from the aforementioned study may not quite correspond to the results of our study owing to how the quantitative measurements were achieved and the difference in metal artifact reduction technique (tin filtration vs VME). In the previous article, only the width of the most prominent metal artifact was measured. Do et al., (2020) compared different energy thresholds and acquisition modes for PCD-CT and EID-CT after scanning a hip prosthesis phantom. Fewer metal artifacts were found in high-energy threshold images compared to low-energy thresholds and the choice of acquisition mode had an influence on the number of artifacts seen in relevant structures such as cortical bone and bone marrow [26]. These findings warrant further investigation in future studies in order to corroborate the findings of this study and to identify the ideal settings for postoperative CI imaging with the novel PCD-CT technology. This study yields some limitations. First, the study has a small sample size and this study is a cadaveric study. Further in vivo studies with larger sample sizes are needed to confirm the results. Another limitation is the use of a nominal Likert scale for the visibility of interelectrode wires.

## 5. Conclusions

The study presents the first results using PCD-CT technology for CI with VME images.

In comparison to EID-CT, the visibility of interelectrode wire sections was significantly higher for PCD-CT and PCD-CT technology leading to larger electrode contact diameter measurements closer to the defined gold standard.

There was no difference in halo artifact diameter between the EID- and PCD-CT for VME images.

## Data Availability

The data presented in this study are available on request from the corresponding author. The data are not publicly available due to ethical restrictions.
